# Impact of Tigecycline on C. difficile Outcomes: Case Series and Propensity-Matched Retrospective Study

**DOI:** 10.1128/aac.00001-22

**Published:** 2022-06-01

**Authors:** Emma C. Phillips, Cirle A. Warren, Jennie Z. Ma, Gregory R. Madden

**Affiliations:** a University of Virginia School of Medicine, Charlottesville, Virginia, USA; b Division of Infectious Diseases & International Health, Department of Medicine, University of Virginia School of Medicine, Charlottesville, Virginia, USA; c Department of Public Health Sciences, University of Virginia Health System, Charlottesville, Virginia, USA

**Keywords:** *Clostridioides difficile*, tigecycline

## Abstract

This case series and propensity-matched cohort study on the use of tigecycline in Clostridioides difficile infection (CDI) evaluated the effect of tigecycline on 30-day mortality. Adjusted for ATLAS Score, hypotension, treatment time period, and serum lactate, tigecycline did not significantly improve 30-day mortality (odds ratio: 0.89; 95% confidence interval: 0.25–3.12; *P = *0.853). A randomized controlled trial is needed to determine efficacy and safety of tigecycline in severe or refractory CDI.

## INTRODUCTION

Clostridioides difficile infection (CDI) remains potentially lethal in an unacceptably large proportion of inpatients (1). Tigecycline has been used off-label as adjunctive treatment in severe or refractory CDI (2); however, there are no randomized controlled trials to date supporting its use. While some case reports (3–5) and limited retrospective analyses suggest higher rates of CDI cure with tigecycline (6), other observational studies have failed to demonstrate any statistically significant benefit while adjusting for confounding factors (7–9), and some suggest increased rates of mortality and colectomy (10). Furthermore, tigecycline does not appear to reduce CDI recurrence (3, 9, 11), and excess all-cause mortality is associated with tigecycline for non-CDI indications (12, 13).

**FIG 1:**
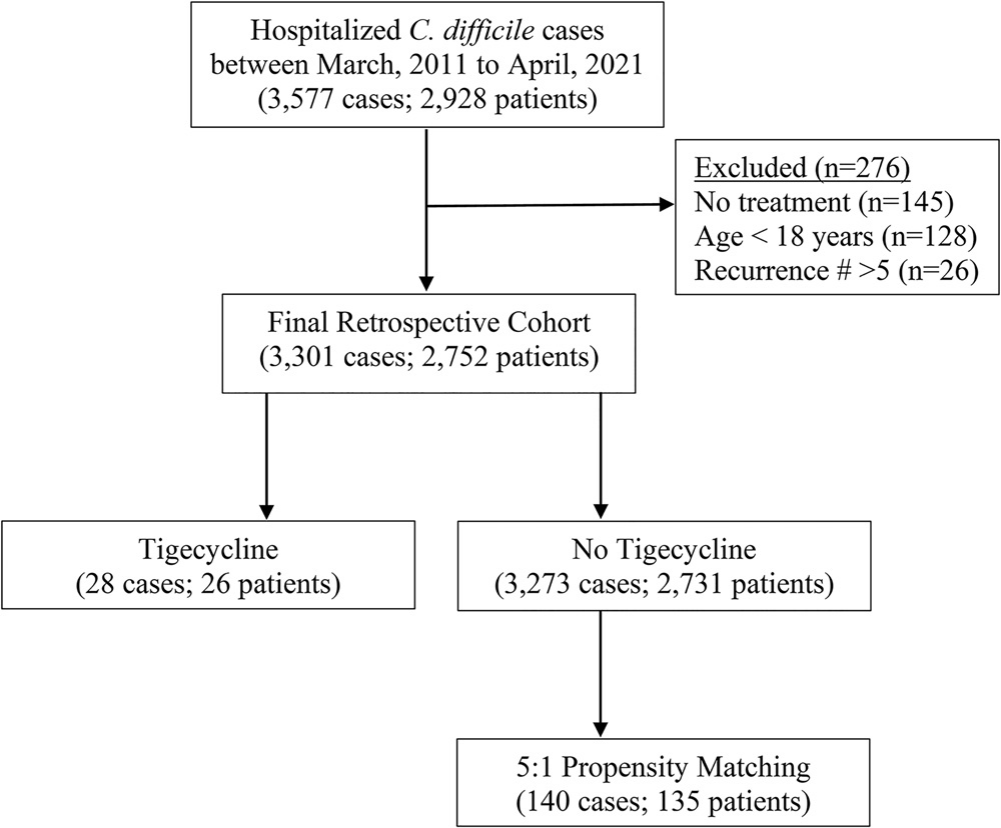
C. difficile infection propensity-matched cohort.

A retrospective case series analysis and propensity-matched cohort study were conducted at University of Virginia (UVA) Hospital to evaluate hospitalized adult patients with C. difficile infection administered >1 dose of tigecycline during treatment. This study received approval from UVA Internal Review Board (no. 20082). Inpatient episodes with a CDI diagnosis (based on positive C. difficile PCR; GeneXpert; Cepheid) were identified between March 2011 and April 2021 (Fig. 1) and stratified into two treatment periods (2011–2016 and 2017–2021) marked by implementation of computerized decision support tool in December 2016 (14) and updated 2017 CDI management guidelines (15). Patients <18 years, with > 5 prior recurrent episodes, or who did not receive active treatment (oral vancomycin, IV/oral metronidazole, or tigecycline) were excluded.

Baseline clinical data, including laboratory measurements within ±48 h of the positive PCR, and outcome data were gathered electronically from the UVA Clinical Data Warehouse. Modified Charlson Comorbidity Index was calculated using International Classification of Diseases coding data (16, 17). ATLAS/Zar Scores were measured at diagnosis (18, 19). Analyses were performed using statistical software R, version 4.0.4 (R Core Team, Vienna, Austria) with ‘comorbidity,’ ‘MatchIt,’ and ‘gee’ packages.

Baseline characteristics of each cohort are in Table 1. In the full cohort, tigecycline-treated patients had significantly higher pressor and antimotility agent use, lower albumin, and higher ATLAS and Zar Scores. A significantly higher proportion of tigecycline cases occurred after 2016. The propensity-matched cohort showed no significant differences in baseline characteristics.

**TABLE 1:** Baseline characteristics of full and propensity-matched CDI cohorts^*a*^

Characteristic	Full cohort			Propensity matched		
	No tigecycline (*N* = 3,273)	Tigecycline (*N* = 28)	*P* value	No tigecycline (*N* = 140)	Tigecycline (*N* = 28)	*P* value
Age						
Mean (SD)	60.7 (16.5)	56.1 (12.9)	0.0704	56.0 (17.8)	56.1 (12.9)	0.982
Gender						
Male	1,643 (50.2%)	18 (64.3%)	0.195	90 (64.3%)	18 (64.3%)	1
Race						
White	2,617 (80.0%)	23 (82.1%)	0.969	122 (87.1%)	23 (82.1%)	0.538
African American	594 (18.1%)	5 (17.9%)		16 (11.4%)	5 (17.9%)	
Asian	15 (0.5%)	0 (0%)		0 (0%)	0 (0%)	
Other	47 (1.4%)	0 (0%)		2 (1.4%)	0 (0%)	
Ethnicity						
Hispanic	36 (1.1%)	0 (0%)	0.966	1 (0.7%)	0 (0%)	0.571
Hypotension					
SBP <90	1,202 (36.7%)	15 (53.6%)	0.1	75 (53.6%)	15 (53.6%)	1
Pressors	314 (9.6%)	8 (28.6%)	**0.00229**	35 (25.0%)	8 (28.6%)	0.874
Fever	1,122 (34.3%)	9 (32.1%)	0.97	63 (45.0%)	9 (32.1%)	0.296
Ileus or megacolon	1,104 (33.7%)	8 (28.6%)	0.708	41 (29.3%)	8 (28.6%)	1
Intensive care unit	385 (11.8%)	5 (17.9%)	0.483	25 (17.9%)	5 (17.9%)	1
NHSN Classification					
CO-CDI	1,189 (36.3%)	6 (21.4%)	0.157	29 (20.7%)	6 (21.4%)	0.904
CO-HCFA-CDI	575 (17.6%)	8 (28.6%)		46 (32.9%)	8 (28.6%)	
HO-CDI	1,509 (46.1%)	14 (50.0%)		65 (46.4%)	14 (50.0%)	
Comorbidities					
CHF	441 (13.5%)	3 (10.7%)	0.714	21 (15.0%)	3 (10.7%)	0.714
PVD	308 (9.4%)	0 (0%)	0.132	15 (10.7%)	0 (0%)	0.132
Dementia	67 (2.0%)	1 (3.6%)	1	0 (0%)	1 (3.6%)	0.384
COPD	563 (17.2%)	5 (17.9%)	1	31 (22.1%)	5 (17.9%)	0.719
Rheum	117 (3.6%)	0 (0%)	0.556	2 (1.4%)	0 (0%)	1
Diabetes	863 (26.4%)	8 (28.6%)	0.962	41 (29.3%)	8 (28.6%)	1
Renal	634 (19.4%)	8 (28.6%)	0.325	45 (32.1%)	8 (28.6%)	0.882
Cancer	717 (21.9%)	9 (32.1%)	0.283	51 (36.4%)	9 (32.1%)	0.829
AIDS	15 (0.5%)	0 (0%)	1	0 (0%)	0 (0%)	1
Charlson Comorbidity Index				
Mean (SD)	1.77 (1.38)	1.68 (1.35)	0.731	2.12 (1.52)	1.68 (1.35)	0.157
Recurrence					
Initial	2.718 (83.0%)	22 (78.6%)	0.0538	102 (72.9%)	22 (78.6%)	0.547
1	371 (11.3%)	3 (10.7%)		25 (17.9%)	3 (10.7%)	
2	105 (3.2%)	2 (7.1%)		6 (4.3%)	2 (7.1%)	
3	47 (1.4%)	0 (0%)		3 (2.1%)	0 (0%)	
4	22 (0.7%)	0 (0%)		3 (2.1%)	0 (0%)	
5	10 (0.3%)	1 (3.6%)		1 (0.7%)	1 (3.6%)	
White blood cell count (cells/μL)				
Mean (SD)	14.4 (10.8)	22.5 (28.5)	0.15	20.9 (17.1)	22.5 (28.5)	0.785
Creatinine (mg/dL)					
Mean (SD)	2.15 (2.29)	2.91 (2.40)	0.112	2.89 (2.31)	2.91 (2.40)	0.972
Albumin (mg/dL)					
Mean (SD)	2.66 (0.712)	2.24 (0.636)	**0.00274**	2.44 (0.707)	2.24 (0.636)	0.164
Lactate (mg/dL)					
Mean (SD)	2.38 (2.19)	3.92 (4.83)	0.182	3.20 (2.87)	3.92 (4.83)	0.541
Non-CDI antibiotics during treatment	1.830 (55.9%)	19 (67.9%)	0.282	94 (67.1%)	19 (67.9%)	1
Immunosuppression	449 (13.7%)	7 (25.0%)	0.148	37 (26.4%)	7 (25.0%)	1
Antimotility use	169 (5.2%)	4 (14.3%)	**<0.001**	14 (10.0%)	4 (14.3%)	0.715
ATLAS Score (0–10)					
Mean (SD)	3.99 (2.10)	5.36 (2.28)	**0.00389**	5.38 (2.16)	5.36 (2.28)	0.964
Zar Score (0–6)					
Mean (SD)	1.67 (1.27)	2.29 (1.49)	**0.00371**	2.26 (1.42)	2.29 (1.49)	0.926
Year						
2011	347 (10.6%)	1 (3.6%)	**<0.001**	4 (2.9%)	1 (3.6%)	0.233
2012	459 (14.0%)	5 (17.9%)		20 (14.3%)	5 (17.9%)	
2013	437 (13.4%)	2 (7.1%)		12 (8.6%)	2 (7.1%)	
2014	385 (11.8%)	3 (10.7%)		12 (8.6%)	3 (10.7%)	
2015	408 (12.5%)	2 (7.1%)		17 (12.1%)	2 (7.1%)	
2016	396 (12.1%)	3 (10.7%)		20 (14.3%)	3 (10.7%)	
2017	258 (7.9%)	0 (0%)		15 (10.7%)	0 (0%)	
2018	209 (6.4%)	3 (10.7%)		17 (12.1%)	3 (10.7%)	
2019	172 (5.3%)	3 (10.7%)		10 (7.1%)	3 (10.7%)	
2020	158 (4.8%)	2 (7.1%)		10 (7.1%)	2 (7.1%)	
2021	44 (1.3%)	4 (14.3%)		3 (2.1%)	4 (14.3%)	

a *n* (%) unless otherwise specified. *P* values calculated using independent-samples *t* tests (continuous variables) and chi-square tests (categorical variables). *P* values in bold-faced type are considered to be significant. Covariates included in the propensity estimation model: age, gender, hypotension, recurrence number, pressors, creatinine, albumin, ATLAS, Zar, leukemoid reaction (white blood cells >30,000 cells/μL), intensive care, immunosuppression, non-CDI antibiotic during treatment, antimotility drug within 7 days, ileus/megacolon, cancer, renal disease, and National Healthcare Safety Network (NHSN) Surveillance definition. SD, standard deviation; SBP, systolic blood pressure; HO-CDI, hospital-onset C. difficile infection; HO-HCFA, hospital-onset health care-facility-associated CDI; CO-CDI, community-onset CDI; CHF, congestive heart failure; PVD, peripheral vascular disease; COPD, chronic obstructive pulmonary disease; Rheum, rheumatologic disease.

The case series was manually compiled by investigator E. C. Phillips using REDCap data capture tools hosted at UVA (20, 21). Cases were stratified into categories of nonsevere, severe, and fulminant infection based on current CDI management guideline criteria (15). Twenty-eight cases of tigecycline treatment were identified among 26 individuals. Seven out of twenty-eight (25%) cases were classified as nonsevere, 12/28 (43%) as severe, and 9/28 (32%) as fulminant infection. Tigecycline was given for an average 7.3 (range: 0.5–27.5; standard deviation: 6.1) days. In the nonsevere/severe groups, tigecycline was used primarily as salvage therapy (Table 2). Mortality was highest in the fulminant group, and recurrence rates were equivalent among surviving patients in the severe and fulminant groups. Tigecycline was used exclusively for CDI in 18/28 (64%) cases, CDI plus another infection in 4/28 (14%) cases, and primarily for another infection (examples include pneumonia, intrabdominal abscess, Enterobacter sepsis, and urinary tract infection) in 6/28 (21%) cases.

**TABLE 2:** Outcomes of tigecycline treatment for CDI from case series

Outcome	Nonsevere infection	Severe infection	Fulminant infection
Avg length of tigecycline therapy	7.4 days (range 0.5–13.6)	7.75 days (range 2.5–20.3)	7.7 days (range 0.5–27.5)
Tigecycline used as initial, salvage, or nondirected therapy^*a*^	Salvage	Salvage	=
In-hospital mortality	2 of 7 (29%)	2 of 12 (17%)	5 of 9 (56%)
90-day mortality	2 of 7 (29%)	2 of 12 (17%)	6 of 9 (67%)
Recurrences at 30 days	0	2	1
Recurrences at 90 days	0	5	1
Total recurrences	5 (in 2/5 [40%] surviving patients)	5 (in 5/10 [50%] surviving patients)	1 (in 1/2 [50%] surviving patients who reached follow-up)

a Initial therapy is defined as tigecycline use within 7 days from day 0: the earliest of the date of positive stool test, the start of directed antimicrobial therapy, or the start of tigecycline therapy. Salvage therapy is defined as tigecycline use after 7 days from day 0. An equals sign indicates that tigecycline was used as initial, salvage, or nondirected therapy in an equal number of cases.

The primary outcome was 30-day all-cause mortality. Secondary outcomes were in-hospital mortality attributable to CDI, colectomy, or diverting ileostomy due to CDI, CDI recurrence, and length of stay. Chart reviews by investigator G. R. Madden identified 130/179 (72.6%) deaths and 15/18 (83.3%) colectomies or ileostomies attributable to CDI. Propensity scores were estimated using a logistic regression model, with tigecycline therapy as the outcome. Nearest neighbor matching was performed at 5:1 control:case ratio to optimize covariate balance and statistical power. The effect of tigecycline on the outcomes of interest was evaluated in the logistic regression, with and without adjusting for baseline characteristics. Repeated CDI episodes were accounted for using the generalized estimating equation method.

Unadjusted 30-day mortality was higher among tigecycline-treated patients (4/28 [14.3%] tigecycline versus 173/3,273 [5.3%] nontigecycline; *P* < 0.001). Compared with propensity-matched controls, mortality in the tigecycline group was not statistically different (4/28 [14.3%] tigecycline versus 12/140 [16.4%]; *P = *1.00). After risk adjustment in the propensity-matched cohort, tigecycline did not significantly improve 30-day mortality (Table 3; odds ratio: 0.89; *P = *0.853); however, this is limited by small case numbers.

**TABLE 3:** Impact of tigecycline from the multivariable logistic regression with generalized estimating equation method^*a*^

*N*_subjects_	161		
Cases	168		
	30-day mortality		
Variable	Odds ratio	95% CI	*P*
Tigecycline	0.89	0.25–3.12	0.853
ATLAS Score	1.33	1.03–1.72	**0.026**
Hypotension	1.93	0.66–5.61	0.227
2017–2021 (vs. 2011–2016)	1.76	0.72–4.31	0.216
Lactate ≥ 2.0 mg/dL	2.54	1.01–6.38	**0.047**

a *P* values in bold-faced type are considered to be significant. CI, confidence interval.

Univariate and multivariable analyses of the secondary outcomes are shown in Table 4. Adjusted coefficients for tigecycline were significantly greater than zero for both total length of stay and length of stay following CDI diagnosis, indicating significantly longer lengths of stay with tigecycline. Colectomy/diverting ileostomy due to CDI, hospital mortality attributable to CDI, and subsequent recurrence were all not significantly associated with tigecycline in the univariate and multivariable analyses.

**TABLE 4:** Propensity-matched cohort: secondary outcomes with tigecycline^*a*^

	Univariate analyses			Multivariable analyses		
Outcome	No tigecycline (*N* = 140)	Tigecycline (*N* = 28)	*P* value	Odds ratio or estimate (LOS)	95% CI	*P* value
Colectomy/diverting ileostomy due to CDI	4 (2.9%)	3 (10.7%)	0.167	4.45^*b*^	0.59–32.4	0.128
Hospital mortality attributable to CDI	21 (15.0%)	5 (17.9%)	0.924	1.42	0.39–5.14	0.594
Subsequent recurrence	30 (21.4%)	7 (25.0%)	0.868	1.19	0.4 −3.28	0.742
Hospital length of stay					
Mean days (SD)	23.1 (27.2)	37.1 (46.3)	0.131	0.71	0.39–1.03	**<0.001**
Hospital length of stay after CDI					
Mean days (SD)	15.2 (20.5)	27.8 (43.5)	0.146	1.08	0.69–1.47	**<0.001**

a *n* (%) unless otherwise specified. *P* values for univariate analyses calculated using chi-square tests or independent-samples *t* tests (length of stay). *P* values in bold-faced type are considered to be significant. For results of multivariable logistic regression (colectomy/ileostomy, attributable mortality, recurrence) and linear regression (total length of stay, length of stay after CDI) with generalized estimating equation method (to adjust for within-subject correlation), all models were adjusted for ATLAS Score, hypotension, time period, and serum lactate. Hospital length of stay calculated between admission/discharge and length of stay after CDI calculated between CDI diagnosis and discharge. Length of stay regression coefficients represent the estimated differences (in days) for the tigecycline group compared with nontigecycline (coefficients significantly greater than zero interpreted as longer length of stay in tigecycline group). LOS, length of stay; CI, confidence interval; SD, standard deviation.

b Generalized estimating equation method could not be applied to colectomy/diverting ileostomy due to low event numbers so ordinary multivariable logistic regression was used.

Although nonsignificant, the C. difficile-associated mortality in the later study period (2017–2021) was higher. This may be in part due to the aforementioned decision support tool, which led to 41% fewer tests and proportionally fewer cases with subclinical infection or colonization; therefore, the proportion of cases in the later period was not only higher, but also likely more severe (14).

There are several potential explanations for why tigecycline may not be effective adjunct therapy in CDI. Although not available clinically, oral tigecycline may be preferable to intravenous administration due to high protein binding in the bloodstream (22). Additionally, the FDA noted that most deaths from early clinical trials were related to progression of an underlying infection, perhaps owing to its bacteriostatic action and/or complex pharmacokinetics in the setting of bacteremic infections, which could be an underrecognized feature in severe CDI (https://www.fda.gov/drugs/drug-safety-and-availability/fda-drug-safety-communication-fda-warns-increased-risk-death-iv-antibacterial-tygacil-tigecycline) (23).

A strength of this study was its large pool of retrospective controls that allowed for robust 5:1 propensity matching; however, small numbers of cases represent a major limitation of the study. Additionally, as an observational study, there may have been bias associated with tigecycline treatment and severe outcomes that may not have been captured; for example, refractoriness of C. difficile-associated diarrhea and treatment response could not be reliably assessed using electronic medical record data. The process of clinical attribution for the secondary outcomes was not blinded to treatment status. Also, median time from CDI diagnosis to first tigecycline administration was 4 days (interquartile range: 6); these outcomes may have already occurred or begun to occur before tigecycline could have had an effect. Finally, tigecycline was utilized for other indications than CDI in several cases.

Tigecycline for adjunctive C. difficile treatment should be carefully weighed against delay in pursuing potentially life-saving aggressive measures such as surgical intervention. A randomized controlled trial is needed to better characterize the role, if any, of tigecycline in the treatment of severe, fulminant, and/or refractory C. difficile infection.
